# Supplementary figures and images for: DagSim: Combining DAG-based model structure with unconstrained data types and relations for flexible, transparent, and modularized data simulation
Source: PLoS One. 2023 Apr 14;18(4):e0284443. doi: 10.1371/journal.pone.0284443 (PMC10104342; doi:10.1371/journal.pone.0284443)

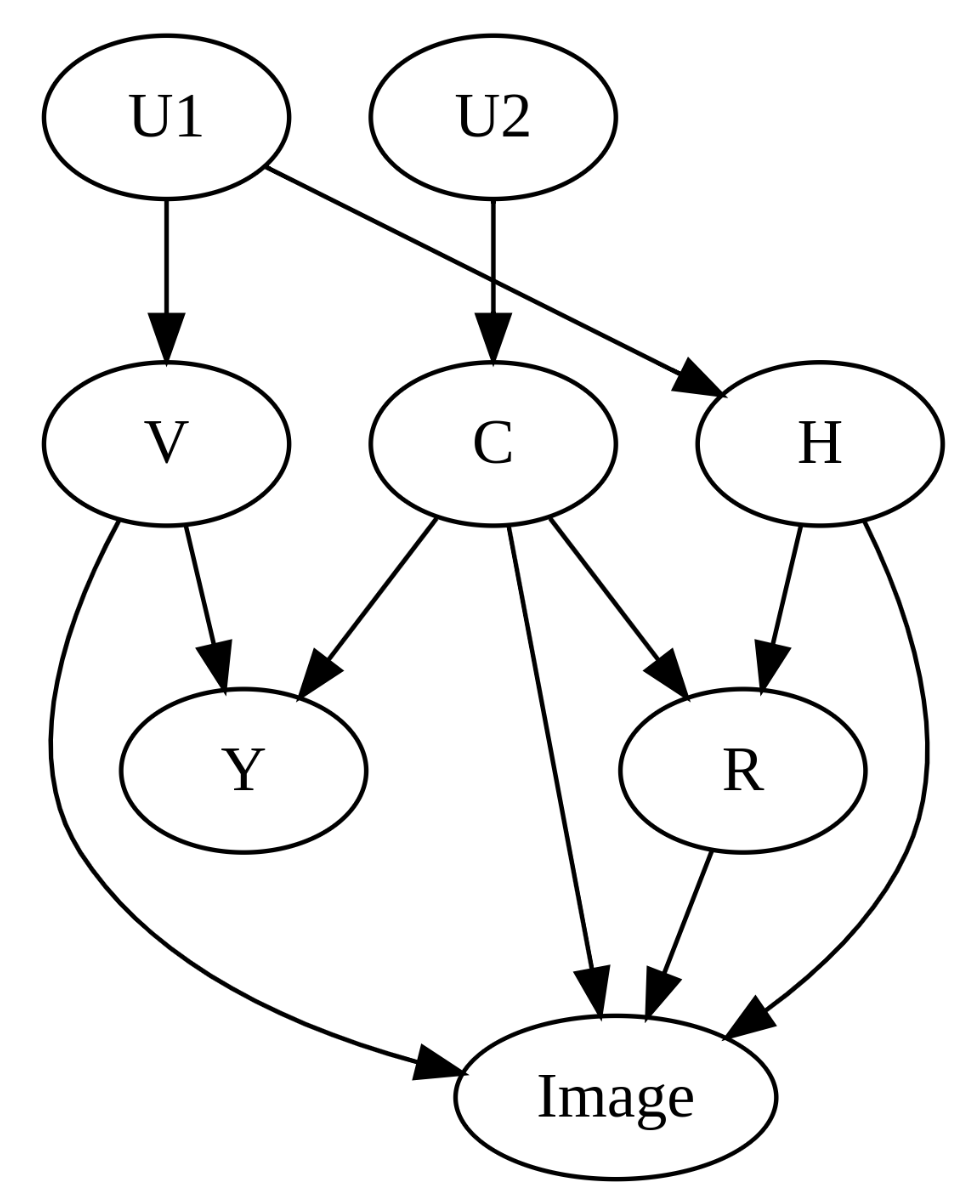

Supplement: S1 Fig — (TIF) [file pone.0284443.s001.tif]

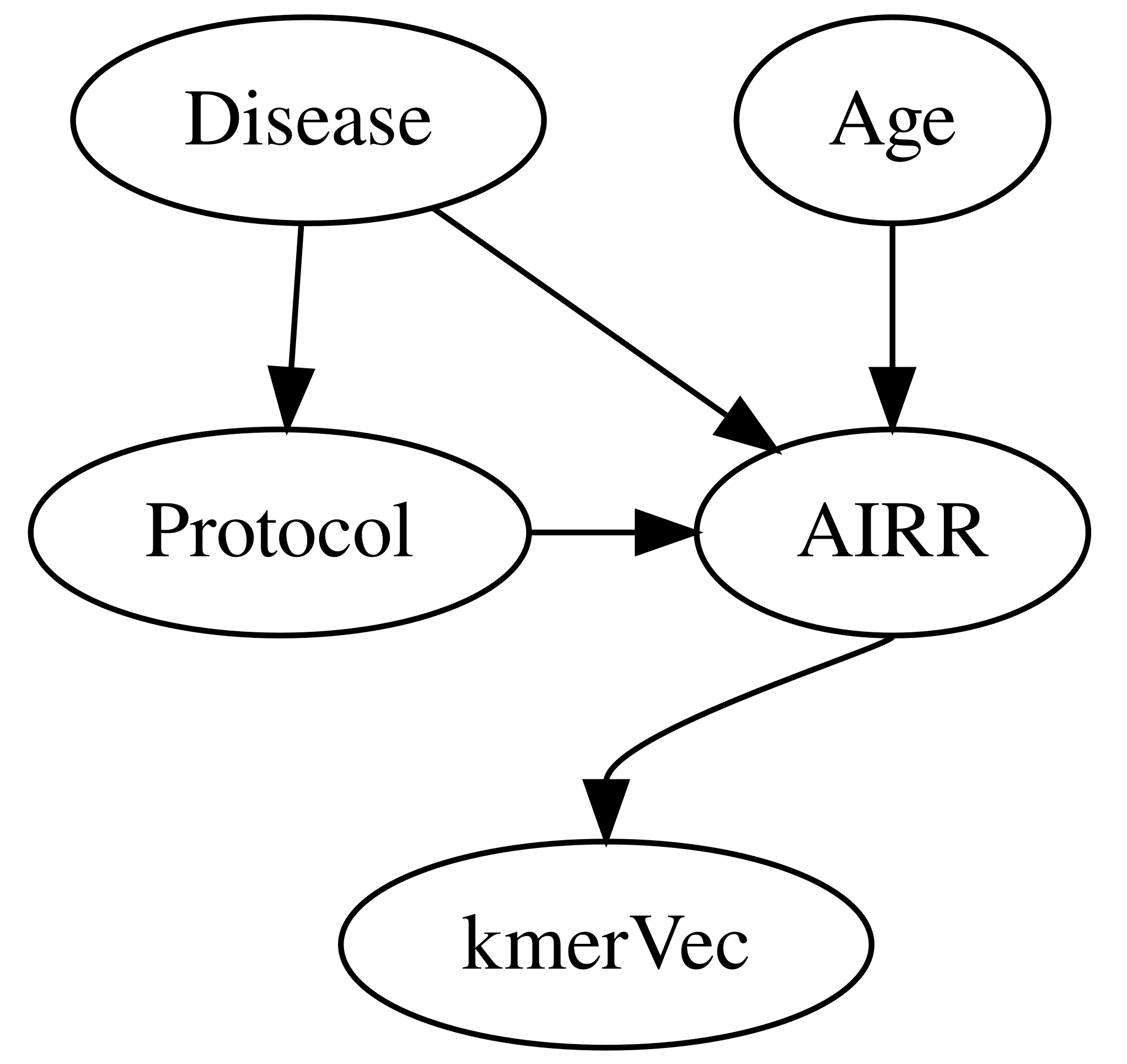

Supplement: S2 Fig — (TIF) [file pone.0284443.s002.tif]
