# Supplementary material for: DagSim: Combining DAG-based model structure with unconstrained data types and relations for flexible, transparent, and modularized data simulation
Source: PLoS One. 2023 Apr 14;18(4):e0284443. doi: 10.1371/journal.pone.0284443 (PMC10104342; doi:10.1371/journal.pone.0284443)
Supplement: Attachment — Submitted filename: Response to Reviewers.pdf [file pone.0284443.s003.pdf]

Reply to the Reviewers

"DagSim: Combining DAG-based model structure with unconstrained data types and relations for flexible, transparent, and modularized data simulation"

Ghadi S. Al Hajj, Johan Pensar, Geir Kjetil Sandve

PLOS ONE

Contents

Introduction **1**

Editor **1**

Reviewer 1 **1**

Reviewer 2 **3**

In this point-by-point response, our response is written in blue. Changes made to the manuscript text are also highlighted in blue and are referred to from the point-by-point response by page number.

Introduction

We would like to express our sincere gratitude to the editor and the reviewers for the time and effort they spent reviewing our manuscript. Their comments have helped us improve this manuscript and thereby make the framework more accessible and beginner-friendly.

Editor

Comment #1

There should be a description section that presents step-by-step development and also the use of a flow chart is encouraged.

Response

We thank the editor for this suggestion which we agree is valuable for the future reader. We have added a new section to the manuscript under the name "Step-by-step example" on page 6 that illustrates the general workflow that one would follow when simulating data using DagSim, demonstrating that with a simple example of simulating text sequences. The corresponding code, YAML snippet, and commands are included in the supplementary material.

Comment #2

Supplementary Material 2 is not available

Response

We thank the editor for pointing out this mistake in the manuscript - we have included all supplementary information in one single document, and now correctly refer to it simply as "Supplementary material".

Reviewer 1

In the manuscript "DagSim: Combining DAG-based model structure with unconstrained data types and relations for flexible, transparent, and modularized data simulation", Ghadi et al. present a python based framework for DAG-based data simulations: DagSim which integrates complex data types and graphical modelling (that is, neither restricted by variable types nor functional forms). More so, the simulation model is defined in the YAML format promoting transparency. The authors demonstrated the framework on two use cases which are available on Github (examples, where basic metadata variables are controlling the shapes in an image and patterns in bio-sequences).

Comment #1

However, they are not clear on the selection criteria they used for the presented current frameworks (are these all the existing tools/packages?):

Response

We thank the reviewer for raising this issue. We would like to point out that we did not set this up as a systematic review, but rather, we aimed to include any framework that is at least tangentially related to the topic of DAG-based simulation, and then described qualitatively and in table form the features of relevance included in these frameworks. We now explicitly state in the legend for Table 1 that it includes "An overview of all established frameworks that to the authors' knowledge offer DAG-based simulation functionalities" to make this clearer.

Comment #2

When simulating simple DAGs (discrete and continuous data), how does dagSim compare to the other simulation frameworks (for example, simMixedDAG and dagR)?

Response

We thank the reviewer for raising this important question. In such a case, it would depend on the functional form that relates the variables to each other. Specifically, it would depend on whether the other frameworks offer such functions, given that all of the other frameworks support only a pre-specified set of functions to use, e.g. polynomials, exponentials, etc. If the functions are simple, then it is most likely that the other frameworks would offer such functions, and in such a case, DagSim would not have an advantage in this respect. We have added a paragraph to the end of Conclusions to clarify this important point: "The examples of simulating shapes in images and patterns in biosequences are but two exemplifications of dagsim's advantages. While existing frameworks also allows to define transparent simulations in settings with standard functional relations between numeric scalars or vectors, only DagSim allows transparency and code modularization on a broad range of settings with complex data types and functional relations. "

Comment #3

Table 1: An overview of the current frameworks that offer simulation functionalities. dagR does not have any simulation utilities, however, Duan et al. 2021 (Reflection on modern methods: understanding bias and data analytical strategies through DAG-based data simulations, https://academic.oup.com/ije/article/50 /6/2091/6272915) demonstrated that dagR can be useful in addressing selection and information bias analysis. Please, clarify if dagitty and dagR doesn't have simulation utilities

Response

We thank the reviewer for pointing out the potentially misleading wording used in the table. We agree that dagR does indeed provide simulation functionality, in particular, the dag.sim/dag.sim2 functions, as also used for the analysis in the "DAG-based data simulation" section of Duan et al). What we formerly meant by simulation utilities is the additional distinctive features, if any, that are provided by a given tool to supplement the core simulation functionality.

Similarly, we agree that the dagitty framework also includes the functions simulateSEM and simulateLogistic that can be used for simulation.

To clear this confusion, we have renamed the ”simulation utilities” column to ”distinctive features”.

Reviewer 2

The authors introduce a novel Direct Acyclic Graph (DAG) - based data simulation Python library called DagSim. They survey existing data simulation tools, discussing them and highlighting a common limitation i.e. that their inputs are limited to numeric variables and specified functional forms. DagSim has the unique advantage of having the capacity to use variables and functional relations of any form. Furthermore, it is modular and easily updatable. An implementation of DagSim has been made available on Github.

Comment #1

Table 1, as provided, is a very useful summary of existing tools, their capabilities, and limitations. I would recommend an additional column in which associated citations for the tools are provided.

Response

We thank the reviewer for the appreciation of the survey performed in this paper. We have added a column to the table that includes the references to each cited tool.

Comment #2

In addition to Figure 1, it would be useful to present a demonstration of an example of the limitation of the other tools' input variable limitations, and DagSim's advantage in processing more complex data types.

Response

Thanks for the suggestion. We have added these two paragraphs to the manuscript, one for each use case, on pages 8 and 9, respectively:

- If this use case was to be performed by any of the existing DAG-based simulation frameworks, then the scalar values would have to be simulated separately using an appropriate DAG. Following that, a separate function that takes as input the variables V, C, R, and H and iterates on all the samples could be used to create the desired image. This would detach the image construction process from the rest of the simulation making the code unnecessarily complicated and less transparent.
- If this use case was to be performed by any of the existing DAG-based simulatThaion frameworks, one would have to use a numeric representation of the sequences, with for example the use of ad hoc of end-of-sequence numeric codes to emulate a set of variable length sequences. As the frameworks also do not support the specification of custom functions, one would need to supplement a DAG-based simulation of baseline sequences (in numeric representation) with post-hoc functions for implanting desired signals.

Comment #3

Pages need to be numbered.

Response

Thanks. Added now.

Comment #4

There is a citation in the Abstract. That needs to be removed.

Response

Thanks. We have removed that citation from the abstract and included it in the introduction instead.

Comment #5

In the last section under "Use", "Additionally, it is known that e.g. the age of a patient leaves a mark on the AIRR (19)..." needs to be corrected

Response

Thanks. We have reformulated the sentence in the manuscript as "Additionally, it has been proposed that immune repertoires become less diverse with age (19) and that experimental protocols introduce particular sequence biases in observed AIRR datasets (20,21)."
